# The Role of the mHealth in the Fight against the Covid-19: Successes and Failures

**DOI:** 10.3390/healthcare9010058

**Published:** 2021-01-08

**Authors:** Daniele Giansanti

**Affiliations:** Centre Tisp, Istituto Superiore di Sanità, Via Regina Elena 299, 00161 Roma, Italy; daniele.giansanti@iss.it; Tel.: +39-06-4990-2701

## 1. The Covid-19: A Pandemic Exploded during the Mobile Technology Era

The spread of the SARS-CoV-2 coronavirus has pushed all affected countries to analyze all of the opportunities offered by current technology, which generated both a high number of solutions and a great debate on their actual ability to face the challenges promoted by pandemic spread. Previously, an epidemic caused by a coronavirus (SARS-CoV), the SARS (Severe Acute Respiratory Syndrome) of 2003, had been addressed. The epidemic lasted over a period of time from November 2002 to July 2003. During the previous pandemic, current mobile technologies were not available and in particular the smartphone as we know it today did not exist.

During each phase of the evolution of the pandemic, mobile technology (*mTech*) has played and is still playing an *important triple role*.

The *first role* is the traditional one played in the field of *digital health* [1,2,3,4,5] by connecting citizens to the health system and providing them with highly innovative technological solutions.

The *second role* is to support teaching, work and relational activities in an exceptional way, allowing social distancing between subjects, such as through messaging and/or video conferencing and/or social network tools [6,7].

The *third role* is specific to this pandemic and consists in providing *mHealth* solutions for controlling and monitoring the spread of the pandemic, such as through App-based solutions for the *digital contact tracing* [8,9].

The Covid-19 pandemic has hit every corner of the planet; however, the access to these technological solutions has not always been and still is not uniform due to the phenomenon called *digital divide* which depends on multifaceted aspects ranging from the lack of access to instrumental and network resources, to cultural and social barriers [10,11] and also to possible forms of communication disability.

## 2. The Traditional Role of the *mHealth* in the Covid-19 Era

During the pandemic, *mHealth*, which rests its foundations on *mTech*, continued to play its traditional role, in a more incisive and impressive way to increase the action of social distancing. In many realities we have gone from an *mHealth* used only in pilot experiences, and/or merely linked to research experiences, to an *mHealth* used in routine clinical applications regulated from every point of view [1] (including reimbursement). This last point has been reached in some cases also thanks to a rapid regulatory update, which often has provided for specific exceptions [2].

The advantages provided by *mHealth* [4,5] for the patient in the pandemic era are the following ones [3]:

1. Promoting a healthy lifestyle and improving the awareness, active participation and motivation of individuals for health care solutions and technologies.
2. Facilitation and speeding up of doctor/patient communication and treatments tailored to the patients anywhere without the need for a stable/fixed workstation.
3. Increase in the autonomy and safety of the patient who can be remotely monitored and located using the *mTech* (e.g., smartphone, smartwatches, wearable sensors, wearabe devices).
4. All the advantages inherited from telemedicine for all actors involved in health care.
5. Improving social distancing without sacrificing the continuity of care.
6. Decreasing, as a consequence of the previous point, the risk of contagion from Sars-CoV-2, through, for example, the use of triages procedures using *mHealth*.

## 3. The Generalized Support of the Mobile Technology

During the pandemic there has been and currently there is an unprecedented use of Apps dedicated to communication for messaging/video. These Apps are mainly used [6,7] in the following sectors:

- Work activity, where there has been a massive introduction of the *smart working tool*.
- Didactics, where there was the introduction of methods of delivery of courses in remote mode.
- Social communication activities to allow the maintenance of relationships in all sectors including the relationships within the family.

Often thanks to these technologies we have witnessed the development and proliferation of spontaneous networks which in fact have provided, where applied, real psychological support to avoid isolation and loneliness [7].

## 4. The New Boundaries Explored by the *mHealth*

During the pandemic, it was possible to explore a new potential of *mHealth* thanks to the availability of technological resources not accessible in the previous SARS pandemic. The key resource is definitely the *smartphone*. In fact, only in 2008 did we gradually witness the development of mobile technologies as we know them today, thanks to the *smartphone*, which has, compared to previous mobile technologies, some peculiar characteristics. The availability of this device was useful for creating *contact tracing* in *digital* form (*DCT*), widely used in epidemiology to allow monitoring and control of the spread of the pandemic. In general, the *smartphone* differs from the basic mobile phone due to the presence of the following features [4,5]:

- The increased memory, a higher computing capacity, a much more advanced data connection capacity due to the presence of dedicated operating systems.
- A great potential for the production and management of multimedia content such as taking high-resolution photos, producing video clips.
- The ability to easily install free and/or paid features and/or applications (Apps).
- The provision of a high-resolution touch screen.
- The ability to use/operate a virtual keyboard to interact with the various functions of the device (from the address book to the notepad), with the web, with the various applications installed and with the so-called social networks.
- The integration with sensors such as accelerometers, gyroscopes, magnetometers, thermometers and even in the most advanced models: photoelectric sensors, laser depth sensors, hall effect sensors, proximity sensors, barometers.
- The possibility of tethering (i.e., providing internet access to other devices through hot spots) over the wireless network, Wi-Fi or Bluetooth, to devices such as other *smartphone*s or mobile phones, laptops or fixed computers.
- The availability of GPS sensors.

Among the above listed *smartphone features* useful in the context of the *DCT* [8,9], we find in particular:

- The capability to find in virtual stores (Google Play and Apple Store) easily to be installed Apps;
- The availability of the functions GPS and Bluetooth and the related evolutions.
- The accessibility to speedy networks and very wide databases.

Virtually almost all governments on the planet have invested energy to build App-based solutions for the *DCT*; some governments creating national Apps for *DCT*s (like Italy), other ones creating local regional Apps (like the USA); some nations carrying out a precise monitoring of the population, based on GPS, even on a mandatory basis; other nations using Bluetooth based protocols, with a purely voluntary membership, to ensure greater respect for privacy [8,9].

## 5. The Obstacles Caused by the *Digital Divide*

An important obstacle to a full and complete use of technologies in the ways described above has in some cases been represented by the *digital divide* [10,11] which is still mainly caused by the following problems:

- *Access to the data network* limited or by the availability of resources in the region or in some cases by political reasons, such as for example due to tensions between ethnic groups and/or groups belonging to different government positions within the same state.
- *Social factors*. Due, for example, to access difficulties in disadvantaged social categories who, even for economic reasons, cannot access these technologies.
- *Cultural factors*. Even within regions with full access to technologies, uneven access to technologies was found due to cultural and training barriers. Certainly the *mobile-born*, for example, have experienced a better ability to adapt than even elderly teachers and elderly doctors. Specifically, with regard to *mobile-born* targeted studies, for example, these will be able to give us information on the role played during the Covid-19 pandemic in eventually breaking down the *digital divide* barrier but also on any other encountered problems (also perspective articles are here strongly needed and welcome).
- *Disabilities*. Disabilities, such as communication disabilities, which generally represent an obstacle in a non-pandemic period to access to technologies, continued to represent an obstacle even during the Covid-19 pandemic.

## 6. Conclusions

The Covid-19 pandemic has created an unprecedented impetus for the development of *mHealth* [1,2,3,4,5]. This development involved both the enhancement and standardization of already consolidated solutions in *digital health* and the exploration of new potentials such as those of the *DCT* [8,9]. In many cases, the simple *mTech* itself has represented a *real lifebuoy* [6,7] both for the continuation of normal activities (working and teaching) and for providing a safety net. However, this has not always happened in a uniform way. The *digital divide* was a cause of this [10,11].

Making a map of these aspects, by means of scientific article contributions is important both to consolidate experiences and to ensure that they are not lost for the future and in particular for the post-pandemic era.
